# Phytochemical and Antioxidant Profile of Pardina Lentil Cultivars from Different Regions of Spain

**DOI:** 10.3390/foods10071629

**Published:** 2021-07-14

**Authors:** Ângela Liberal, Ângela Fernandes, Maria Inês Dias, José Pinela, Ana María Vivar-Quintana, Isabel C. F. R. Ferreira, Lillian Barros

**Affiliations:** 1 Centro de Investigação de Montanha (CIMO), Instituto Politécnico de Bragança, Campus de Santa Apolónia, 5300-253 Bragança, Portugal; angela.liberal@ipb.pt (Â.L.); maria.ines@ipb.pt (M.I.D.); jpinela@ipb.pt (J.P.); iferreira@ipb.pt (I.C.F.R.F.); lillian@ipb.pt (L.B.); 2 Food Technology, Higher Polytechnic School of Zamora, Universidad de Salamanca, 37008 Salamanca, Spain

**Keywords:** *Lens culinaris*, Pardina, nutritional value, chemical composition, kaempferol derivates, antioxidant activity

## Abstract

Lentils (*Lens culinaris* spp.) are an important food consumed worldwide given their high protein, fiber, mineral, and phytochemical contents, and can be used as a potential source of good nutrition for many people. With the purpose of valuing the Pardina variety, the quality brand from a protected geographical indication “Lenteja de Tierra de Campos”, a full assessment of the nutritional, chemical, and antioxidant properties of 34 samples from this variety was carried out. Besides its actual rich nutritional profile, three phenolic compounds by high performance liquid chromatography equipped with photodiode array detection-mass (HPLC-DAD-ESI/MS) were identified (kaempferol derivatives) with slight differences between them in all extracts. Sucrose by high-performance liquid chromatography with a refraction index detector (HPLC-RI) and citric acid by ultra-fast liquid chromatography coupled with a photodiode array detector (UFLC-PDA) were the major identified sugar and organic acid components, respectively, as well as α-tocopherol and γ-tocopherol isoforms (HPLC-fluorescence). Additionally, all the extracts presented excellent antioxidant activity by the oxidative hemolysis inhibition assay (OxHLIA/TBARS). Briefly, Pardina lentils from this quality brand are a good source of nutritional and chemical components and should therefore be included in a balanced diet.

## 1. Introduction

Food culture is becoming more diverse with changes in society, which has been engaged in the search for functional foods that can not only integrate vegetarian and vegan diets, but also meet the nutritional needs of those suffering from food intolerance problems, while protecting animal welfare and the environment [1,2]. The commitment of the scientific community to the search for nutraceuticals in functional foods of plant origin was crucial in its discovery and in the analyses of its properties [3,4,5,6]. In this search, prebiotics and probiotics, selective and viable microorganisms that positively influence human health in addition to their nutritional properties, were also identified as a part of a wide variety of legumes [7,8]. For this reason, legumes have been the subject of several scientific studies focused on their chemical and nutritional profiles, bioactive properties, and the beneficial health effects derived from their consumption. In particular, legume seeds play an essential role in human diet and in various physiological and metabolic processes as they are exceptional sources of protein, minerals, vitamins, and bioactive compounds [9], features that make them the main foundation of food in emerging nations [10].

There are several varieties of pulses growing worldwide, for instance, beans, chickpeas, cowpeas, and lentils, which present different nutritional and chemical profiles [11]. Among these, lentils (*Lens culinaris* L.), also known as red dhal, masur, or split peas, are an edible pulse considered an essential and inexpensive source of dietary protein in unindustrialized countries [11], frequently consumed as a full grain or in a decorticated and fragmented shape with other cereals such as rice [12]. Although lentils are a relatively long-lasting plant grown around the world, the main manufacturing countries are Canada, Turkey, the USA, Australia, and India, contributing to over three quarters of the world’s total production [13]. Among legumes, and in addition to being a source of plant-based protein, lentils hold notable amounts of fibers and minerals as well as phytochemicals including phenolic acids, flavanols, saponins, phytic acid, and condensed tannins, presenting good antioxidant assets [12,14]. Furthermore, the presence of β-glucans in this type of pulse promotes short-range satiety and a low glycemic response, contributing to body weight maintenance [15]. Lentils have also been found to be an interesting source of prebiotic compounds [13] that help in the preservation of the intestinal microbiota and prevent gut-associated diseases [7]. These and other features suggest that lentil consumption may be associated with health benefits such as a reduced risk of cardiovascular illness, cancer, diabetes, osteoporosis, hypertension, gastrointestinal disorders, and adrenal ailments, and a reduction in low-density lipoprotein (LDL) cholesterol [16,17,18,19,20]. Current research suggests that protein hydrolysates and peptides, together with dietary fiber and their colonic fermentation products (i.e., short chain fatty acids, SCFA) that may influence gut and colon well-being, may be in control of some of these health incomes [21,22]. Micronutrients such as phenolics have also revealed solid antioxidant properties in lentils. However, despite these outcomes, there is no agreement on the precise bioactive constituent(s) responsible for these beneficial health incomes. Although lentils’ nutritional and chemical characteristics are well known, environmental factors, soil composition, irrigation, and other features may disturb the composition and amounts of soluble carbohydrates in plants, as they do with other seed storage components [23].

Castilla y León is the place in Spain where the greatest quantity and varieties of legumes are produced, among which is the Pardina lentil “Lenteja de Tierra de Campos” [21]. Protected geographical indication (PGI) “Lenteja de Tierra de Campos” is a quality brand that occupies some of the northwestern provinces of Castilla y León. This region is located in the northern sub-plateau of the Iberian Peninsula and its orography, multiple and distinct, gives rise to a great variety of climates, characterized by strict winters and warm summers, with brief spring and autumn periods. The use of products from PGI guarantees the quality and authenticity of the same as well as contributing to a sustainable rural development, namely through the use of regional varieties, and preserving the genetic resources and rural diversity [22].

Therefore, the present study aims to analyze the nutritional and chemical profiles as well as the antioxidant capacity of different cultivars of the Pardina lentil “Lenteja de Tierra de Campos” grown in different regions of Spain, contributing to its characterization and inclusion in a daily balanced diet.

## 2. Materials and Methods

### 2.1. Samples

Thirty-four different cultivars of Pardina lentils (*Lens culinaris*, sub-species microsperma, family Europeae) from the PGI “Lenteja de Tierra de Campos” were collected in different production areas within its territory, all belonging to the 2018 campaign. This quality brand occupies part of the northwestern provinces of Castilla y León: León, Palencia, Valladolid, and Zamora, located in the northern sub-plateau of the Iberian Peninsula. The samples were ground with skin in a Foss Knifetec™ 1095 mill with temperature control (23 °C). The cotyledons are yellow, and the outer skin is brown or brown and mottled or black-veined, which can cover the entire lentil. The minimum size of the grains was 35 mm, although up to 4% of smaller lentils was allowed.

### 2.2. Standards and Reagents

HPLC-grade acetonitrile was obtained from Merck KgaA (Darmstadt, Germany). Standards of melezitose, fatty acids methyl ester (FAME, reference standard mixture 3747885-U), Trolox (6-hydroxy-2,5,7,8-tetramethylchroman-2-carboxylic acid), and 2,2′-azobis(2-methylpropionamidine) dihydrochloride (AAPH) were purchased from Sigma Chemical Co. (St. Louis, MO, USA); tocol standard (50 mg/mL) was acquired from Matreya, Pleasant Gap (PA, USA), and the phenolic compound standards were acquired from Extrasynthese (Genay, France). Ethanol and all other chemicals and solvents were of analytical grade and purchased from common sources. Water was treated in a Milli-Q water purification system (TGI Pure Water Systems, Greenville, SC, USA)

### 2.3. Nutritional Value

The samples were analyzed for nutritional composition (protein, fat, carbohydrates, and ash) according to the Official Methods of Analysis of AOAC [24]. Crude protein was estimated by the macro-Kjeldahl method (N × 6.25) via an automatic distillation and titration unit (model Pro-Nitro-A, JP Selecta, Barcelona, Spain). Soxhlet extraction was employed to access the crude fat with petroleum ether for 7 h. The ash content was determined by incineration at 550 ± 10 °C [25,26]. Total carbohydrates were calculated by difference: total carbohydrates (g/100 g) = 100 − (fat + g ash + g proteins). Total energetic value was calculated according to the Atwater system using the formula: Energy (kcal/100 g fresh weight (fw)) = 4 × (g protein + g carbohydrates) + 9 × (g fat). The nutritional value was expressed as g/100g fw. All analyses were carried out in triplicate.

### 2.4. Chemical Characterization

#### 2.4.1. Free Sugars

Free sugar extraction from the ground samples was performed according to Barros et al. [27]. The compounds were identified by high-performance liquid chromatography with a refraction index detector (HPLC-RI; Knauer, Smartline 1000 and Smartline 2300 systems, respectively), as previously described by the authors. Peak identification was carried out by comparisons of their relative retention time (Rt) with an authentic standard. Quantification was finished using melezitose as an internal standard (IS; Sigma-Aldrich, St. Louis, MO, USA), and with calibration curves constructed from authentic standards. The results were managed in Clarity software (Data Apex, Prague, Czech Republic) and expressed in g per 100 g of fw.

#### 2.4.2. Organic Acids

Organic acids were determined following a previously described procedure and optimized by the authors [1]. In brief, samples (~1.5 g) were extracted by stirring with 25 mL of metaphosphoric acid (25 °C at 60 g) for 25 min, and subsequently filtered through Whatman no. 4 paper (diam. 12.5 cm). The assessment was achieved by ultra-fast liquid chromatography coupled with a photodiode array detector (UFLC-PDA; Shimadzu Corporation, Kyoto, Japan). The compound separation was carried out in a C18 SphereClone (Phenomenex, Alcobendas, Spain) reverse phase column (5 µm, 250 × 4.6 mm id) thermostated at 35 °C using 3.6 mM sulfuric acid (0.02%) solution as an eluent at a flow rate of 0.8 mL/min. The identification was carried out by comparing the chromatograms obtained for the analyzed samples with those obtained using commercial standards. The quantification of the compounds was completed by relating the peak areas, recorded at 215 nm, with the calibration curves obtained with commercial standards for each compound. The results were expressed in g per 100 g of fw.

#### 2.4.3. Tocopherols

Tocopherols were determined following a procedure previously described by Barros et al. [27]. The formerly described HPLC system was used, coupled to a fluorescence detector (FP-2020; Jasco, Japan) automated for excitation at 290 nm and emission at 330 nm. Separation of the tocopherol isoforms was attained using a normal phase column of Polyamide II (250 mm × 4.6 mm i.d.) from YMC Waters (Japan), functioning at 30 °C. The mobile phase used was a mixture of hexane and ethyl acetate (7:3, *v*/*v*), with a flow rate of 1 mL/min and an injection volume of 20 µL. Quantification was founded on the response of the fluorescence signal, using the IS method (IS solution in hexane: tocol; 50 μg/mL; 400 μL) and by chromatographic comparison with standards. Tocol (Matreya, Pleasant Gap, State College, PA, USA) was used as an IS. The results were expressed in mg per 100 g of fw.

#### 2.4.4. Fatty Acids

Fatty acid methyl esters (FAME) were investigated after the trans-esterification of the lipid fraction, obtained through Soxhlet extraction, as previously described [28], and determined by gas-liquid chromatography with flame ionization detection, using a YOUNG IN Chromass 6500 GC System instrument equipped with a split/splitless injector, a flame ionization detector (FID), and a Zebron-Fame column. Fatty acid identification and quantification were performed by comparing the relative retention times of FAME peaks from samples with standards (standard mixture 47885-U, Sigma, St. Louis, MO, USA), and the results were recorded and processed using the Software Clarity DataApex 4.0 software (Prague, Czech Republic) and expressed in relative percentage of each fatty acid.

### 2.5. Non-Nutrient Composition

#### 2.5.1. Extract Preparation

For decoctions, each sample (~3 g) was boiled with 100 mL of distilled water for 5 min in a heating plate and then filtrated through Whatman filter paper no. 4. The obtained decoctions were frozen and lyophilized to obtain a dried extract (FreeZone 4.5, Labconco, Kansas City, MO, USA) [25].

#### 2.5.2. Phenolic Compounds

Phenolic compounds were determined in lyophilized decoction extracts, which were re-dissolved in distillated water to a final concentration of 10 mg/mL, filtered through 0.22 μm disposable filter disks. Chromatographic separation of the compounds was achieved with a Waters Spherisorb S3 ODS-2 C18 column (3 µm, 4.6 mm × 150 mm, Waters, Milford, MA, USA), operating at 35 °C. The elution solvents, working in the gradient, were 0.1% formic acid in water and acetonitrile. Finally, to detect MS in negative mode, a Linear Ion Trap LTQ XL mass spectrometer (ThermoFinnigan, San Jose, CA, USA) equipped with an electrospray ionization source (ESI) was used. The identification of phenolic compounds was based on chromatographic performance, spectra, and UV-Vis masses by comparison with standard compounds or the data previously described in the literature, using the Xcalibur^®^ software (ThermoFinnigan, San Jose, CA, USA). Quantitative analysis of the recognized compounds was completed using calibration curves based on the UV signal of the standard compounds. When commercial standards were not accessible, the calibration curves of the most similar standards were used. The operating conditions were previously described in detail by Bessada et al. [29] as well as the identification and quantification procedures. The results are expressed in mg per g of fw.

### 2.6. Antioxidant Activity Evaluation

#### 2.6.1. Thiobarbituric Acid Reactive Substances (TBARS)

For the antioxidant activity assays, the lyophilized decoction extracts were re-dissolved in water, and subjected to dilutions from 5 to 0.0781 mg/mL. Lipid peroxidation inhibition in porcine (*Sus scrofa*) brain homogenates was evaluated by the decrease in TBARS; the color intensity of malondialdehyde–thiobarbituric acid (MDA–TBA) was measured by its absorbance at 532 nm; the inhibition ratio (%) was calculated using the following formula: [(A − B)/A] × 100%, where A and B are the absorbance of the control and the sample solutions, respectively [30]. The results were expressed in EC_50_ values (µg/mL, sample concentration providing 50% of antioxidant activity).

#### 2.6.2. Oxidative Hemolysis Inhibition Assay (OxHLIA)

The anti-hemolytic activity of the lyophilized decoctions was evaluated by the oxidative hemolysis inhibition assay (OxHLIA), as described in detail by Lockowandt et al. [30]. An erythrocyte solution (2.8%, *v*/*v*; 200 µL) was mixed with 400 µL of either extract solution (0.0938–3 mg/mL PBS), PBS (control), or water (for complete hemolysis). After pre-incubation at 37 °C for 10 min with shaking, AAPH (200 μL, 160 mM in PBS, from Sigma-Aldrich) was added, and the optical density was measured at 690 nm every ~10 min in a microplate reader (Bio-Tek Instruments, ELX800) until complete hemolysis [30]. Trolox was used as a positive control. The results were expressed as IC_50_ values (µg/mL) at Δ*t* of 60 and 120 min, which translated the extract concentration required to keep 50% of the erythrocyte population intact for 60 and 120 min.

### 2.7. Statistical Analysis

For all the experiments, thirty-four samples were analyzed, and the antioxidant assays were carried out in triplicate. The results are expressed as mean values ± standard deviation (SD). The differences between the different samples were analyzed using an analysis of variance (ANOVA) based on the Tukey test, with α = 0.05, coupled with Welch’s statistic, to classify the statistical differences between the different parameters evaluated. IBM SPSS Statistics for Windows, version 22.0, was Corp., Armonk, NY, USA). The correlations between parameters were studied by Pearson (IBM correlation (*p* < 0.05)).

## 3. Results and Discussion

### 3.1. Nutritional Composition

The results attained for the macronutrients are presented in Table 1. Carbohydrates were the main macronutrients found in all samples (62.21 to 67.84 g/100 g fw), followed by proteins (20.62 to 25.94 g/100 g fw), ash (2.07 to 2.83 94 g/100 g fw), and fat, which presented the lowest values (0.82 to 1.22 g/100 g fw). Regarding the carbohydrates, 46% corresponded to starch and 11% to dietary fiber for lentils in the 2018 campaign (data not analyzed in our samples). The results reached for the nutritional parameters of the studied lentils, mainly its low fat and high protein and carbohydrate contents, make them essential in low-caloric diets. Additionally, the total ash is indicative of good micronutrient concentration, which is vital at various levels and involved in several mechanisms in the human organism. These outcomes are within a similar range to those from a study performed by Ramdath et al. [31], who investigated the proximate composition and nutritional properties of 20 Canadian freeze-dried, ground, and cooked lentils. Their study identified similar amounts in the evaluated parameters, with carbohydrates presented as the major macronutrient in all samples, as in our study. Similar results have also been described for lentils from Pakistan [32] and Iran [33]. Regarding fat content, values may range from 0.52 g/100 g, as described for Spanish lentils [34], to values higher than 3 g/100 g, described for Mexican cultivars [20]. Although many studies have characterized the nutritional composition of lentils, the data are not always comparable. The proximate composition of lentils can be affected by factors such as variety, climatic conditions, water, and fertilizer application [33]. Thus, the Crimson, Blaze, Redwing, and Robin varieties, all grown in Canada, showed significant differences in protein content. Even for each of these varieties, differences were found in their composition depending on the producing farm, with two different protein levels found for each variety [33]. Thus, for lentil protein content, concentrations between 23.30–25.55% have been described for Pakistan cultivars [33], between 19.9 and 26.8% in Spanish cultivars [33] and between 28.8 and 30.60% for Chinese ones [33]. In this study, all of the analyzed seeds belonged to the same variety and were grown in the same region under the same agro-geoclimatic conditions. For this reason, the compositional parameters analyzed showed very narrow ranges of variability. These results show that the assurance of a certified nutritional quality is possible within protected geographical indications.

### 3.2. Chemical Composition

Free sugars were also analyzed, and the results are presented in Table 2. Sucrose and raffinose were the only two sugars identified in all 34 samples of the Pardina lentils, with sucrose presenting the highest values (0.90 to 1.14 g/100 g fw), and raffinose concentration ranging between 0.15 and 0.24 g/100 g fw, suggesting a low-glycemic index associated with these pulses. Regarding the total sugar content, the found values oscillated between 1.09 and 1.36 g/100 g fw. No statistically significant variations (*p* > 0.05) were found between the mean values achieved for the identified compounds in all samples. Johnson et al. [35] also investigated the sugar profile of lentils from different countries, detecting, in turn, sucrose, raffinose + stachyose and verbascose in all of the samples analyzed. In their study, raffinose + stachyose were quantified together, and their amounts fluctuated from 3.31 g/100 g fw in Lebanese lentils to 4.80 g/100 g fw in Moroccan ones. In our study, although quantified alone, raffinose was detected in very inferior amounts compared to the above study. Additionally, Tahir et al. [23], in a study carried out on Canadian lentils where the effects of environmental conditions on the content and composition of soluble carbohydrates in lentil seeds were evaluated, identified superior amounts of sucrose (1.22–1.67 g/100 g lentil meal) with statistically significative differences between cultivars from different regions. Their study proves that the local precipitation positively influences the amount of sucrose found in the studied lentils, with higher concentrations found in cultivars grown in regions with higher precipitation rates.

Regarding organic acids in all of the analyzed samples, oxalic, shikimic, and citric acids were identified (Table 3), with the latter standing out due to its high concentrations, fluctuating between 10.51 and 18.06 g/100 g fw. Due to its extraordinary physicochemical assets and environmentally benign nature, citric acid is reported as a preservative, emulsifier, flavorant, sequestrant, and as a buffering mediator commonly used in several industries, especially in food, beverage, pharmaceutical and nutraceutical products [36]. Oxalic and shikimic acids were found in lower amounts (0.20 to 0.31 g/100g fw and 0.03 to 0.10 g/100g fw, respectively). The occurrence of organic acids in lentils has been sparsely investigated. However, Morales et al. [37], in a study performed with lentil flours, spotted quite a few compounds of nutritional and bioactive attention, specifically organic acids. In all samples of raw lentil-formulated flours, oxalic, tartaric, quinic, malic, and fumaric acids were identified, with malic acid being found in higher concentrations (0.98 to 1.24 g/100 g fw). However, the comparison of results from lentils and their flours must take into account the fact that in flour preparation, certain treatments may be involved such as boiling, roasting, and germination, which can alter the nutritional and chemical composition of these pulses.

With respect to the assessment of tocopherols, the isoforms α and γ were identified in all 34 samples analyzed (Table 4), with γ-tocopherol standing out as an isoform detected in higher concentrations (2.45 to 4.28 mg/100g fw). γ-Tocopherol embodies a strong anti-inflammatory and antioxidant agent; thus, due to the identification of high amounts of this tocopherol isoform in food, it is suggested to be responsible for the reduced risk of developing cardiovascular diseases, among others [38]. α-Tocopherol mean concentrations (0.21 to 0.36 mg/100 fw) similarly did not show significant differences between the analyzed samples. Our results were consistent with those obtained by Boschin and Arnoldi [39], whose study also pointed toward γ-tocopherol as the predominant isoform in lentils, with similar amounts of total tocopherol concentration. As was noted for the other analyzed parameters, different types of cultivars have a strong influence on the total and individual tocopherol content [40]. Zhang et al. [41] performed a study with 20 different cultivars of Canadian lentils to identify, among other parameters, the tocopherol profile present in these samples. The results showed that the total tocopherol amounts of the different cultivars varied between 64.4 μg/g and 37.4 μg/g dw, with the cultivars Greenland and Plato presenting the highest and lowest concentrations, respectively, thus emphasizing the influence of different cultivars on the chemical composition of lentils.

An evaluation of the fatty acids profile was also performed, and 21 compounds were identified in all the analyzed samples (Table A1 in Appendix A, Table 4). The major compounds presented are C18:2n6 (linoleic acid, 40.04 and 45.66%), followed by C18:1n9 (oleic acid, 13.08 and 19.58%), C18:3n3 (α-linolenic, 12.79 to 15.85%), and C16:0 (palmitic acid, 12.63 and 16.06%), which stand out as the most important fatty acids, showing no significant differences (*p* > 0.05) among the different samples being studied. Regarding their classification, the polyunsaturated fatty acids (PUFA) lodged the uppermost concentrations (53.1–60.95%), followed by saturated fatty acids (SFA; 20.54–27.16%) and, finally, monounsaturated fatty acids (MUFA; 15.46–27.16%).

Our results are, in part, in agreement with a study performed by Zhang et al. [42], who indicated that linoleic acid was the predominant fatty acid present in the analyzed red and green lentil varieties, which also showed good amounts of α-linolenic fatty acid, which, together, made PUFAs the predominant form identified. Additionally, similar percentages of palmitic acid were identified by the authors. On the other hand, Zia-Ul-Haq et al. [32], in a study performed on four lentil breeding lines grown in Pakistan, identified unsaturated fatty acids as the major fatty acids in all cultivars. Once again, the influence of different cultivars, genotypes, and geographical origins of lentils on its fatty acid profile was clear. However, with respect to individual fatty acids in lentils, the authors did not identify significant differences depending on cultivar or climatic conditions [32].

### 3.3. Phenolic Compounds

The tentative identification of the phenolic compounds found in decocted extracts of the Pardina lentil “Lenteja de Tierra de Campos” as well as the retention times (Rt), maximum absorbance (λ_max_), pseudomolecular ion ([M-H]^−^), and the main ion fragments (MS^2^) of each phenolic compound, are described in Table 5. Three phenolic compounds were identified in all samples, apart from sample 5, where only kaempferol-deoxyhexoside-hexoside isomer I was present. The phenolic composition of lentils has already been studied by other authors [46,47], who reported a considerable number of compounds (31 and 35, respectively), while our study only identifies three. Previous studies carried out on Pardina lentils [45] showed the presence of hydroxycinnamic and hydroxybenzoic acids (protocatechuic acid, *p*-hydroxybenzoic acid, *trans*-*p*-coumaroyl-malic acid, trans-*p*-coumaroyl-glycolic acid, *trans*-*p*-coumaric acid, cis-*p*-coumaric acid, and *trans*-ferulic acid), as well as flavonols and flavones (myricetin-3-*O*-rhamnoside, luteolin- 7-*O*-glucoside, apigenin-7-*O*-apiofuranosyl glucoside, apigenin-7-*O*-glucoside, luteolin glycoside, quercetin- 3-*O*-rutinoside, and luteolin). The differences between previous studies and the results of this paper could be related to the processing method applied. Once in decoction extracts, samples were subjected to high temperatures, which are known to degrade bioactive compounds, such as phenolic compounds. As far as the authors are concerned, extraction by decoction with water has not been used previously in lentils, which is a novel and complementary contribution to the study of their phenolic composition. Furthermore, the compounds identified here have not been previously described in lentils. For this reason, the authors consider it an interesting contribution to study phenolic compounds in lentils using the water decoction method

Peaks 1 and 2 ([M-H]^−^ at *m*/*z* 901), were tentatively identified as kaempferol-deoxyhexoside-hexoside isomers I and II, respectively, by relating their retention time, UV spectra, and mass fragmentation outlines to the accessible commercial standards. Peak 1 exhibit a base peak at *m*/*z* 593 and peak 2 presented an identical chromatographic performance, leading to the identification of the isomers I and II, respectively. Yeo and Shahidi [45], in a study conducted on hulls from different lentil varieties, identified a peak showing a deprotonated ion [M-H]^−^ at 593 and a fragment ion at *m*/*z* 285, suggesting its tentative identification as a kaempferol derivative. A similar compound was described in other legumes, such as quinoa, by Pilco-Quesada et al. [48]. Peak 3 ([M-H]^−^ at *m*/*z* 755) tentatively identified as kaempferol-*O*-hexoside-*O*-deoxyhexoside-hexoside, were previously identified in Pardina lentils from Badajoz, Spain, and reported by Aguilera et al. [47], exhibiting MS^2^ fragments at *m*/*z* 593 and *m*/*z* 285. On the other hand, Zou et al. [49] observed two pseudomolecular ions at *m*/*z* 901 and 755 in whole Morton lentils, which were tentatively identified as kaempferol tetraglucoside and kaempferol triglucoside, respectively. Kaempferol and kaempferol glycosides were linked to antioxidant, cardioprotective, anticancer, anti-inflammatory and neuroprotective properties [50] and are, therefore, compounds of great interest from the health perspective. Furthermore, kaempferol has been linked to various anticarcinogenic properties [51,52], showing that a high kaempferol intake can reduce the risk of cancer [53].

The quantification data of the phenolic compounds present in the analyzed samples are shown in Table 6. The phenolic profile displayed by the 34 samples in the study was very similar, both quantitatively and qualitatively, with very similar amounts of each compound between samples, which could be explained by the fact that all the samples are from the same region of Spain (Castilla y Léon), and from a PGI, and are therefore subject to similar environmental factors and soil compositions. The total phenolic compounds of the 34 samples analyzed ranged between 30 and 158 μg/g fw, showing no significant differences (*p* > 0.05) among them. The dissolution of phenolic compounds in the freeze-dried decoction extracts was carried out with water, which may explain the small concentrations found for the phenolic compounds identified.

### 3.4. Bioactive Properties

The decocted lyophilized extracts of the 34 samples in the study were used to evaluate their antioxidant properties through their capacity to avoid lipid peroxidation of porcine brain tissues and hemolysis of sheep blood cells, and the results are shown in Table 7. Regarding the TBARS assay, lentil extracts showed good antioxidant activity, presenting EC_50_ values between 211 and 228 μg/mL. Regarding the OxHLIA assay, the extract concentration required to protect half of the erythrocyte population from the hemolytic action (IC_50_) triggered by the oxidative agent for 120 min ranged from 178 µg/mL to 1312 µg/mL, showing no significant differences between samples (*p* > 0.05) which, in spite of being higher than that required for Trolox, was still an acceptable outcome for a natural extract. Once again, these results highlight the high antioxidant activity of the extract, and validate the major potential of these pulses to be applied for antioxidant determinations such as for food engineering. This antioxidant action may be due to the incidence of good tocopherol totals, mainly γ-tocopherol, and organic acids such as citric acid in our samples. The correlations between these parameters and antioxidant activity were studied by Pearson correlation (*p* < 0.05). The parameters analyzed (tocopherols and organic acids) showed no significant correlation with antioxidant activity, neither individually nor in terms of their total content. Only the concentration of α-tocopherol showed a significant correlation with antioxidant activity, although, contrary to expectations, this correlation was negative (r = −0.397).

There are several methods used to evaluate the antioxidant capacity of an extract but most of them are chemical assays, founded on the scavenging of free radicals. An example of this is the 2,2-diphenyl-1-picrylhydrazyl (DPPH) assay, which measures the scavenging ability of antioxidants toward a DPPH radical. Tang et al. [38] assessed the antioxidant activity of red and green lentils using the DPPH assay, and the results expressed good antioxidant activity, mainly due to the presence of carotenoids and tocopherols, which seem to cooperate synergistically. Aguilera et al. [47], using the oxygen radical absorbance capacity (ORAC) assay, a method that associates both inhibition time and degree of inhibition with a single amount, showed good antioxidant activity in raw lentils (66.97 µmol Trolox/g) when compared to other common legumes such as chickpeas, green peas, and yellow peas. It is thus evident that, regardless of the test used to measure antioxidant activity, lentils are shown to be efficient in this field.

## 4. Conclusions

The Pardina lentil is an important source of nutritional and chemical compounds, showing that it is a variety of high nutritional value. Furthermore, the different production areas analyzed in this study have shown that there are no significant differences in their nutritional parameters, evidencing the homogeneity of the lentils covered by the PGI “Lenteja Tierra de Campos”. Our study allowed for the identification of different components such as sugars, organic acids, tocopherols, unsaturated fatty acids, and phenolic compounds, whose presence has been associated with several health benefits such as antioxidant properties, also assessed in this study, with satisfactory outcomes. The nutritional and chemical configuration of this variety of lentil makes it a valuable and suitable food to be introduced to a balanced diet with low-glycemic index, and suitable for the development of new plant-based food products with better nutritional and chemical qualities. Along this line, the characterization of the carbohydrate composition in this variety of lentils is particularly important due to its influence on the glycemic index. This is an element of the research work that should be addressed in the future. Regarding its composition in phenolic compounds, our results revealed significant differences compared to other studies, with the identification of only three compounds in the samples under analysis. These results seem to be associated with the extraction method utilized. However, since the objective of our study was related to the characterization of the Pardina lentil variety from a PGI in the way that it would be consumed, the extraction method used was considered, by us, to be the most appropriate for the study in question. Further studies should be carried out to compare the extraction methodology used in this study with different extraction solvents.

## Figures and Tables

**Table 1:** Nutritional value (g/100 g fw) and energetic value (kcal/100 g fw) of the studied Pardina lentil “Lenteja de Tierra de Campos” (mean ± SD, *n* = 3).

Sample *^1^	Moisture	Fat	Proteins	Ash	Carbohydrates
1	9.39 ± 0.08 ^c^	0.95 ± 0.05 ^klmn^	22.70 ± 0.4 ^kl^	2.50 ± 0.03 ^hij^	64.50 ± 0.2 ^fg^
2	4.91 ± 0.01 ^s^	1.04 ± 0.04 ^fgh^	23.90 ± 0.6 ^g^	2.69 ± 0.03 ^bc^	67.50 ± 0.5 ^ab^
3	9.00 ± 0.1 ^de^	0.88 ± 0.05 ^qrs^	23.88 ± 0.09 ^g^	2.60 ± 0.1 ^cdef^	63.60 ± 0.2 ^ijk^
4	6.09 ± 0.09 ^qr^	1.08 ± 0.03 ^def^	24.80 ± 0.1 ^e^	2.54 ± 0.04 ^fghi^	65.53 ± 0.02 ^e^
5	7.65 ± 0.01 ^l^	0.96 ± 0.05 ^klm^	25.28 ± 0.07 ^bcd^	2.80 ± 0.1 ^a^	63.30 ± 0.1 ^jklm^
6	6.40 ± 0.1 ^op^	0.97 ± 0.02 ^klm^	25.11 ± 0.08 ^cde^	2.80 ± 0.1 ^a^	64.70 ± 0.2 ^f^
7	7.53 ± 0.03 ^l^	1.08 ± 0.01 ^de^	25.50 ± 0.1 ^bc^	2.60 ± 0.1 ^defg^	63.30 ± 0.1 ^jkl^
8	6.73 ± 0.08 ^mn^	1.10 ± 0.02 ^cd^	24.87 ± 0.01 ^de^	2.57 ± 0.02 ^fgh^	64.73 ± 0.02 ^f^
9	6.70 ± 0.4 ^mn^	1.10 ± 0.03 ^cd^	23.30 ± 0.6 ^hi^	2.70 ± 0.1 ^b^	66.20 ± 0.6 ^cd^
10	6.73 ± 0.01 ^mn^	1.15 ± 0.02 ^b^	23.20 ± 0.1 ^ij^	2.45 ± 0.02 ^jkl^	66.50 ± 0.1 ^c^
11	7.97 ± 0.03 ^k^	1.22 ± 0.06 ^a^	25.40 ± 0.7 ^bc^	2.40 ± 0.1 ^m^	63.10 ± 0.4 ^lmn^
12	8.00 ± 0.5 ^k^	1.14 ± 0.02 ^bc^	25.60 ± 0.4 ^ab^	2.90 ± 0.2 ^bcd^	62.60 ± 0.5 ^no^
13	7.50 ± 0.3 ^l^	1.15 ± 0.01 ^b^	22.00 ± 0.2 ^m^	2.40 ± 0.1 ^lm^	66.99 ± 0.03 ^b^
14	9.80 ± 0.1 ^b^	0.90 ± 0.01 ^opqr^	24.00 ± 0.2 ^fg^	2.50 ± 0.1 ^jkl^	62.90 ± 0.3 ^lmn^
15	6.70 ± 0.2 ^mn^	1.01 ± 0.04 ^hij^	25.90 ± 0.1 ^a^	2.60 ± 0.06 ^efg^	63.70 ± 0. 2^hijk^
16	9.30 ± 0.3 ^c^	0.91 ± 0.06 ^nopq^	23.20 ± 0.1 ^ij^	2.53 ± 0.06 ^ghi^	64.00 ± 0.1 ^ghi^
17	6.30 ± 0.1 ^pq^	1.05 ± 0.05 ^efg^	23.80 ± 0.1 ^g^	2.62 ± 0.01 ^efg^	66.20 ± 0.1 ^cd^
18	6.90 ± 0.09 ^m^	1.05 ± 0.04 ^efg^	22.60 ± 0.5 ^l^	2.44 ± 0.05 ^jkl^	67.00 ± 0.3 ^b^
19	5.99 ± 0.01 ^r^	1.04 ± 0.01 ^efgh^	23.10 ± 0.2 ^ijk^	2.62 ± 0.05 ^cdef^	67.30 ± 0.1 ^b^
20	8.40 ± 0.2 ^hi^	0.98 ± 0.02 ^jkl^	22.50 ± 0.3 ^l^	2.10 ± 0.02 ^q^	66.10 ± 0.1 ^cd^
21	8.50 ± 0.1 ^gh^	0.82 ± 0.06 ^s^	21.10 ± 0.2 ^n^	2.10 ± 0.1 ^q^	67.50 ± 0.2 ^ab^
22	9.77 ± 0.01 ^b^	0.94 ± 0.01 ^lmno^	23.90 ± 0.5 ^g^	2.21 ± 0.04 ^no^	63.20 ± 0.4 ^klm^
23	9.76 ± 0.01 ^b^	1.01 ± 0.01 ^hij^	24.80 ± 0.1 ^e^	2.28 ± 0.04 ^n^	62.20 ± 0.1 ^o^
24	6.6 ± 0.1 ^no^	1.04 ± 0.04 ^efgh^	25.50 ± 0.6 ^bc^	2.63 ± 0.02 ^bcdef^	64.20 ± 0.6 ^gh^
25	10.10 ± 0.4 ^a^	0.93 ± 0.02 ^mnop^	22.90 ± 0.2 ^ijk^	2.30 ± 0.1 ^no^	63.80 ± 0.1 ^hij^
26	9.20 ± 0.2 ^cd^	0.95 ± 0.01 ^klm^	22.80 ± 0.3 ^jkl^	2.10 ± 0.1 ^q^	64.90 ± 0.4 ^f^
27	8.70 ± 0.1 ^fg^	0.99 ± 0.04 ^ijk^	25.10 ± 0.6 ^cde^	2.50 ± 0.1 ^klm^	62.80 ± 0.3 ^mn^
28	6.40 ± 0.4 ^p^	0.96 ± 0.04 ^klm^	24.30 ± 0.2 ^f^	2.30 ± 0.1 ^n^	66.10 ± 0.2 ^cd^
29	8.30 ± 0.2 ^hi^	1.03 ± 0.03 ^ghi^	20.60 ± 0.6 ^o^	2.18 ± 0.01 ^op^	67.80 ± 0.5 ^a^
30	7.59 ± 0.04 ^l^	0.85 ± 0.03 ^s^	23.67 ± 0.01 ^gh^	2.13 ± 0.03 ^pq^	65.76 ± 0.03 ^de^
31	8.01 ± 0.04 ^jk^	0.87 ± 0.03 ^rs^	23.92 ± 0.02 ^fg^	2.70 ± 0.1 ^bcde^	64.53 ± 0.01 ^fg^
32	9.30 ± 0.6 ^c^	0.93 ± 0.04 ^mnop^	21.40 ± 0.6 ^n^	2.82 ± 0.02 ^a^	65.56 ± 0.04 ^e^
33	8.80 ± 0.3 ^ef^	0.89 ± 0.04 ^pqr^	23.80 ± 0.6 ^g^	2.50 ± 0.1 ^ij^	64.00 ± 0.7 ^hi^
34	8.25 ± 0.08 ^ij^	0.88 ± 0.05 ^qrs^	24.80 ± 0.7 ^c^	2.50 ± 0.1 ^ijk^	63.60 ± 0.5 ^ijk^

^1^ Different letter in the same column shows significant difference between means according to Tukey’s HSD test (*p* < 0.05). * *Lens culinaris*, sub-species microsperma, family Europeae.

**Table 2:** Composition regarding sugars (g/100 g fw) of the studied Pardina lentil “Lenteja de Tierra de Campos” (mean ± SD, *n* = 3).

	Free Sugars		
Sample *^1^	Sucrose	Raffinose	Total Sugars
1	0.91 ± 0.01 ^c^	0.23 ± 0.01 ^klmn^	1.13 ± 0.01 ^kl^
2	1.04 ± 0.01 ^s^	0.20 ± 0.01 ^fgh^	1.24 ± 0.02 ^g^
3	0.95 ± 0.01 ^de^	0.19 ± 0.01 ^qrs^	1.14 ± 0.01 ^g^
4	1.03 ± 0.01 ^qr^	0.19 ± 0.01 ^def^	1.22 ± 0.01 ^e^
5	1.09 ± 0.01 ^l^	0.21 ± 0.00^klm^	1.30 ± 0.01 ^bcd^
6	1.10 ± 0.01 ^op^	0.23 ± 0.01 ^klm^	1.33 ± 0.01 ^cde^
7	1.06 ± 0.01 ^l^	0.23 ± 0.01 ^de^	1.29 ± 0.02 ^bc^
8	1.08 ± 0.01 ^mn^	0.20 ± 0.01 ^cd^	1.28 ± 0.01 ^de^
9	1.03 ± 0.01 ^mn^	0.19 ± 0.01 ^cd^	1.22 ± 0.01 ^hi^
10	1.12 ± 0.01 ^mn^	0.19 ± 0.01 ^b^	1.31 ± 0.02 ^ij^
11	1.08 ± 0.01 ^k^	0.23 ± 0.01 ^a^	1.31 ± 0.01 ^bc^
12	1.11 ± 0.01 ^k^	0.23 ± 0.01 ^bc^	1.33 ± 0.01 ^ab^
13	0.92 ± 0.01 ^l^	0.20 ± 0.01 ^b^	1.13 ± 0.01 ^m^
14	0.90 ± 0.01 ^b^	0.22 ± 0.01 ^opqr^	1.12 ± 0.01 ^fg^
15	1.03 ± 0.01 ^mn^	0.19 ± 0.01 ^hij^	1.22 ± 0.01 ^a^
16	0.91 ± 0.01 ^c^	0.22 ± 0.02 ^nopq^	1.13 ± 0.02 ^ij^
17	1.03 ± 0.01 ^pq^	0.18 ± 0.01 ^efg^	1.22 ± 0.01 ^g^
18	1.11 ± 0.01 ^m^	0.18 ± 0.01 ^efg^	1.30 ± 0.02 ^l^
19	1.12 ± 0.01 ^l^	0.24 ± 0.01 ^efgh^	1.36 ± 0.02 ^ijk^
20	1.07 ± 0.0 ^hi^	0.23 ± 0.01 ^jkl^	1.29 ± 0.01 ^l^
21	1.10 ± 0.01 ^gh^	0.18 ± 0.01 ^s^	1.28 ± 0.01 ^n^
22	0.99 ± 0.01 ^b^	0.18 ± 0.01 ^lmno^	1.17 ± 0.01 ^g^
23	0.95 ± 0.01 ^b^	0.19 ± 0.01 ^hij^	1.14 ± 0.01 ^e^
24	0.94 ± 0.01 ^no^	0.19 ± 0.01 ^efgh^	1.12 ± 0.01 ^bc^
25	0.92 ± 0.04 ^a^	0.18 ± 0.01 ^mnop^	1.10 ± 0.03 ^ijk^
26	1.00 ± 0.01 ^cd^	0.23 ± 0.01 ^klm^	1.23 ± 0.01 ^jkl^
27	0.91 ± 0.01 ^fg^	0.18 ± 0.01 ^ijk^	1.09 ± 0.01 ^cde^
28	1.09 ± 0.03 ^p^	0.19 ± 0.01 ^klm^	1.28 ± 0.03 ^f^
29	1.14 ± 0.01 ^hi^	0.15 ± 0.01 ^ghi^	1.29 ± 0.01 ^o^
30	0.93 ± 0.01 ^l^	0.19 ± 0.01 ^s^	1.12 ± 0.01 ^gh^
31	0.92 ± 0.01 ^jk^	0.22 ± 0.01 ^rs^	1.14 ± 0.01 ^gh^
32	1.00 ± 0.01 ^c^	0.22 ± 0.01 ^mnop^	1.21 ± 0.01 ^n^
33	1.00 ± 0.01 ^ef^	0.23 ± 0.01 ^pqr^	1.23 ± 0.01 ^g^
34	1.11 ± 0.01 ^ij^	0.23 ± 0.01 ^qrs^	1.35 ± 0.01 ^c^

^1^ Different letter in the same column shows significant difference between means according to Tukey’s HSD test (*p* < 0.05). * *Lens culinaris*, sub-species microsperma, family Europeae.

**Table 3:** Composition regarding organic acids (g/100 g fw) of the studied Pardina lentil “Lenteja de Tierra de Campos” (mean ± SD, *n* = 3).

	Organic Acids			
Sample *^1^	Oxalic Acid	Shikimic Acid	Citric Acid	Total Organic Acids
1	0.26 ± 0.01 ^hjkl^	0.06 ± 0.01 ^ef^	15.30 ± 0.1 ^e^	15.60 ± 0.1 ^f^
2	0.27 ± 0.01 ^efghj^	0.10 ± 0.01 ^a^	18.10 ± 0.1 ^a^	18.40 ± 0.1 ^b^
3	0.24 ± 0.01 ^mnop^	0.08 ± 0.01 ^b^	12.60 ± 0.3 ^kl^	12.90 ± 0.4 ^lmn^
4	0.20 ± 0.01 ^q^	0.07 ± 0.01 ^d^	12.20 ± 0.5 ^m^	12.50 ± 0.5 ^o^
5	0.27 ± 0.01 ^efghj^	0.07 ± 0.01 ^c^	13.50 ± 0.4 ^g^	13.80 ± 0.4 ^h^
6	0.24 ± 0.03 ^op^	0.07 ± 0.01 ^cd^	13.08 ± 0.02 ^ij^	13.39 ± 0.01 ^jk^
7	0.27 ± 0.01 ^fghj^	0.08 ± 0.01 ^b^	13.81 ± 0.06 ^f^	14.20 ± 0.1 ^g^
8	0.25 ± 0.01 ^lmn^	0.06 ± 0.01	11.32 ± 0.01 ^o^	11.63 ± 0.01 ^q^
9	0.20 ± 0.01 ^q^	0.06 ± 0.01 ^fg^	11.00 ± 0.2 ^p^	11.30 ± 0.2 ^r^
10	0.25 ± 0.01 ^klm^	0.04 ± 0.01 ^k^	10.51 ± 0.01 ^q^	10.81 ± 0.02 ^s^
11	0.26 ± 0.01 ^ghjk^	0.05 ± 0.01 ^hi^	12.82 ± 0.01 ^jk^	13.14 ± 0.01 ^kl^
12	0.25 ± 0.01 ^klmn^	0.05 ± 0.01 ^j^	12.60 ± 0.1 ^kl^	12.90 ± 0.1 ^lmn^
13	0.20 ± 0.01 ^q^	0.07 ± 0.01 ^d^	13.20 ± 0.1 ^hi^	13.40 ± 0.1 ^ij^
14	0.28 ± 0.01 ^cd^	0.05 ± 0.01 ^hi^	13.22 ± 0.03 ^ghi^	13.56 ± 0.04 ^hij^
15	0.28 ± 0.01 ^def^	0.06 ± 0.01 ^ef^	12.60 ± 0.1 ^kl^	12.90 ± 0.1 ^lm^
16	0.23 ± 0.01 ^p^	0.05 ± 0.01 ^h^	12.60 ± 0.1 ^kl^	12.90 ± 0.1 ^lm^
17	0.30 ± 0.01 ^ab^	0.05 ± 0.01 ^gh^	12.70 ± 0.3 ^k^	13.10 ± 0.3 ^l^
18	0.31 ± 0.01 ^a^	0.07 ± 0.01 ^cde^	15.10 ± 0.1 ^e^	15.40 ± 0.1 ^f^
19	0.29 ± 0.01 ^bc^	0.1 0 ± 0.01 ^a^	15.95 ± 0.01 ^d^	16.34 ± 0.01 ^de^
20	0.28 ± 0.01 ^def^	0.07 ± 0.01 ^c^	16.00 ± 0.05 ^d^	16.40 ± 0.1 ^de^
21	0.28 ± 0.01 ^de^	0.06 ± 0.01 ^ef^	16.99 ± 0.03 ^b^	17.33 ± 0.03 ^b^
22	0.24 ± 0.01 ^nop^	0.03 ± 0.01 ^m^	15.90 ± 0.1 ^d^	16.20 ± 0.1 ^e^
23	0.24 ± 0.01 ^mnop^	0.06 ± 0.01 ^f^	16.20 ± 0.3 ^d^	16.50 ± 0.3 ^d^
24	0.27 ± 0.01 ^efghj^	0.05 ± 0.01 ^h^	16.50 ± 0.1 ^c^	16.90 ± 0.1 ^c^
25	0.21 ± 0.01 ^q^	0.05 ± 0.01 ^hi^	13.21 ± 0.02 ^ghi^	13.47 ± 0.1 ^ij^
26	0.25 ± 0.01 ^lmn^	0.04 ± 0.01 ^l^	12.30 ± 0.1 ^m^	12.53 ± 0.04 ^o^
27	0.27 ± 0.01 ^defgh^	0.03 ± 0.01 ^m^	12.22 ± 0.04 ^m^	12.53 ± 0.04 ^o^
28	0.27 ± 0.01 ^defgh^	0.05 ± 0.01 ^hij^	14.00 ± 0.1 ^gh^	14.00 ± 0.1 ^hii^
29	0.26 ± 0.01 ^jklm^	0.05 ± 0.01 ^j^	11.60 ± 0.7^n^	11.90 ± 0.7^p^
30	0.27 ± 0.02 ^dfg^	0.06 ± 0.01 ^f^	12.30 ± 0.1 ^m^	12.60 ± 0.1 ^no^
31	0.27 ± 0.02 ^defgh^	0.07 ± 0.01 ^cd^	13.40 ± 0.1 ^gh^	13.70 ± 0.1 ^hi^
32	0.25 ± 0.01 ^mno^	0.05 ± 0.01 ^ij^	12.39 ± 0.03 ^lm^	12.68 ± 0.03 ^mno^
33	0.27 ± 0.01 ^efgh^	0.06 ± 0.01 ^fg^	12.70 ± 0.2 ^kl^	13.00 ± 0.2 ^l^
34	0.25 ± 0.01 ^klmn^	0.03 ± 0.01 ^m^	11.47 ± 0.01 ^no^	11.75 ± 0.01 ^pq^

^1^ Different letters in the same column shows significant differences between means according to Tukey’s HSD test (*p* < 0.05). * *Lens culinaris*, sub-species microsperma, family Europeae.

**Table 4:** Composition regarding tocopherols (mg/100 g fw) and fatty acid composition (%) of the studied Pardina lentil “Lenteja de Tierra de Campos” (%) (mean ± SD; *n* = 3). The most abundant fatty acids and groups of fatty acids are presented.

Sample *^1^	Tocopherols			Fatty Acids				Groups of Fatty Acids		
	*α*-Tocopherol	*γ*-Tocopherol	Total Tocopherols	C16:0	C18:1n9c	C18:2n6c	C18:3n3	SFA	MUFA	PUFA
1	0.28 ± 0.01 ^fgh^	3.89 ± 0.03 ^ijk^	4.17 ± 0.04 ^gh^	14.14 ± 0.01 ^hijk^	15.12 ± 0.04 ^fgh^	44.08 ± 0.06 ^ghi^	14.11 ± 0.07 ^kl^	25.2 ± 0.1 ^e^	16.6 ± 0.1 ^qr^	58.2 ± 0.1 ^m^
2	0.32 ± 0.01 ^bcd^	3.88 ± 0.01 ^ijk^	4.21 ± 0.01 ^fg^	15.93 ± 0.04 ^ab^	14.08 ± 0.02 ^l^	45.13 ± 0.01 ^bc^	14.84 ± 0.07 ^de^	24.6 ± 0.1 ^hij^	24.6 ± 0.1 ^u^	60.0 ± 0.1 ^cd^
3	0.29 ± 0.01 ^defg^	3.98 ± 0.04 ^fg^	4.28 ± 0.04 ^ef^	14.31 ± 0.02 ^ghi^	14.2 ± 0.2 ^l^	44.52 ± 0.01 ^ef^	15.41 ± 0.04 ^b^	24.5 ± 0.1 ^ij^	15.6 ± 0.1 ^u^	59.9 ± 0.1 ^d^
4	0.29 ± 0.02^defg^	4.07 ± 0.01 ^de^	4.36 ± 0.01 ^d^	15.3 ± 0.3 ^cde^	15.7 ± 0.2 ^cde^	44.2 ± 0.2 ^fgh^	14.24 ± 0.06 ^ijk^	24.6 ± 0.4 ^hij^	17.0 ± 0.2 ^no^	58.5 ± 0.2 ^kl^
5	0.35 ± 0.01 ^ab^	4.15 ± 0.03 ^bc^	4.50 ± 0.01 ^b^	14.2 ± 0.2 ^hij^	16.0 ± 0.1 ^c^	43.8 ± 0.2 ^ijk^	13.19 ± 0.06 ^pqrs^	25.2 ± 0.3 ^ef^	17.9 ± 0.1 ^f^	57.0 ± 0.3 ^r^
6	0.32 ± 0.01 ^cde^	3.90 ± 0.07 ^hijk^	4.22 ± 0.08 ^fg^	15.24 ± 0.07 ^cde^	14.3 ± 0.1 ^kl^	43.30 ± 0.04 ^lm^	14.16 ± 0.04 ^jk^	26.2 ± 0.1 ^b^	16.4 ± 0.2 ^st^	57.5 ± 0.1 ^op^
7	0.23 ± 0.06 ^lmn^	4.16 ± 0.05 ^bc^	4.39 ± 0.01 ^d^	14.0 ± 0.1 ^hijk^	14.15 ± 0.05 ^l^	42.76 ± 0.09 ^n^	15.42 ± 0.03 ^b^	25.1 ± 0.1 ^ef^	16.8 ± 0.1 ^pq^	58.2 ± 0.1 ^m^
8	0.28 ± 0.03 ^fgh^	4.13 ± 0.06 ^bcd^	4.42 ± 0.04 ^cd^	15.5 ± 0.3 ^bcd^	13.65 ± 0.07 ^m^	43.99 ± 0.02 ^ghi^	15.37 ± 0.01 ^b^	24.1 ± 0.1 ^lm^	16.6 ± 0.2 ^qr^	59.4 ± 0.1 ^gh^
9	0.27 ± 0.02 ^ghij^	4.10 ± 0.06 ^cd^	4.37 ± 0.04 ^d^	16.1 ± 0.1 ^a^	13.08 ± 0.08 ^n^	45.27 ± 0.06 ^bc^	14.43 ± 0.01 ^ghij^	23.9 ± 0.1 ^m^	16.4 ± 0.1 ^st^	59.7 ± 0.1 ^ef^
10	0.23 ± 0.04 ^lmn^	4.00 ± 0.09 ^ef^	4.23 ± 0.05 ^efg^	15.37 ± 0.09 ^bcd^	14.7 ± 0.3 ^ijk^	43.4 ± 0.2 ^klm^	14.8 ± 0.4 ^def^	24.1 ± 0.3 ^klm^	17.7 ± 0.3 ^fg^	58.2 ± 0.6 ^m^
11	0.24 ± 0.02 ^ijklmn^	4.11 ± 0.04 ^bcd^	4.36 ± 0.02 ^d^	15.17 ± 0.07 ^cde^	14.49 ± 0.07 ^jkl^	44.7 ± 0.3 ^de^	15.74 ± 0.05 ^a^	22.4 ± 0.1 ^tu^	17.2 ± 0.1 ^klm^	60.4 ± 0.2 ^b^
12	0.36 ± 0.02 ^a^	4.28 ± 0.04 ^a^	4.65 ± 0.06 ^a^	14.96 ± 0.04 ^def^	14.2 ± 0.1 ^l^	45.1 ± 0.2 ^bc^	15.85 ± 0.03 ^a^	22.2 ± 0.3 ^u^	16.8 ± 0.1 ^op^	60.9 ± 0.2 ^a^
13	0.27 ± 0.09 ^ghi^	3.95 ± 0.09 ^fghi^	4.23 ± 0.18 ^efg^	13.42 ± 0.09 ^lm^	15.29 ± 0.05 ^efg^	44.17 ± 0.02 ^fgh^	14.60 ± 0.03 ^efgh^	22.8 ± 0.1 ^qrs^	18.4 ± 0.1 ^e^	58.8 ± 0.1 ^i^
14	0.31 ± 0.01 ^cdef^	4.18 ± 0.08 ^b^	4.50 ± 0.08 ^bc^	14.2 ± 0.3 ^hij^	15.44 ± 0.06 ^def^	43.45 ± 0.03 ^klm^	15.25 ± 0.05 ^bc^	22.9 ± 0.1 ^pqr^	18.4 ± 0.1 ^e^	58.7 ± 0.1 ^ij^
15	0.28 ± 0.07 ^efgh^	3.84 ± 0.04 ^k^	4.12 ± 0.1 ^h^	15.4 ± 0.2 ^bcd^	14.97 ± 0.02 ^ghi^	44.2 ± 0.3 ^fgh^	13.8 ± 0.1 ^lm^	24.4 ± 0.3 ^jk^	17.6 ± 0.1 ^ghi^	58.1 ± 0.4 ^m^
16	0.34 ± 0.04 ^abc^	3.97 ± 0.02 ^fgh^	4.31 ± 0.02 ^e^	15.9 ± 0.1^ab^	14.95 ± 0.08 ^ghi^	43.8 ± 0.3 ^ijk^	14.76 ± 0.05 ^def^	24.3 ± 0.1 ^jkl^	17.1 ± 0.1 ^lmn^	58.6 ± 0.2 ^jk^
17	0.24 ± 0.02 ^ijklm^	3.87 ± 0.05 ^jk^	4.12 ± 0.07 ^h^	14.5 ± 0.5 ^fgh^	14.8 ± 0.2 ^hij^	44.11 ± 0.01 ^ghi^	14.16 ± 0.07 ^jk^	24.8 ± 0.3 ^gh^	17.0 ± 0.2 ^nop^	58.3 ± 0.1 ^lm^
18	0.26 ± 0.05 ^ghijk^	3.51 ± 0.05 ^m^	3.77 ± 0.01 ^j^	15.0 ± 0.2 ^def^	13.3 ± 0.2 ^mn^	44.5 ± 0.3 ^ef^	14.4 ± 0.1 ^ghijk^	24.9 ± 0.2 ^fg^	16.2 ± 0.1 ^t^	58.9 ± 0.1 ^i^
19	0.23 ± 0.03 ^klmn^	2.5 ± 0.2 ^s^	2.7 ± 0.2 ^q^	13.9 ± 0.2 ^ijkl^	14.4 ± 0.1 ^jkl^	44.99 ± 0.08 ^cd^	14.63 ± 0.03 ^efg^	23.0 ± 0.1 ^opq^	17.4 ± 0.1 ^ijk^	59.6 ± 0.1 ^f^
20	0.22 ± 0.01 ^lmn^	2.6 ± 0.2 ^r^	2.9 ± 0.2 ^p^	13.99 ± 0.01 ^hijk^	15.4 ± 0.6 ^def^	45.4 ± 0.1 ^ab^	14.48 ± 0.04 ^fghi^	22.8 ± 0.3 ^qrs^	17.3 ± 0.5 ^jk^	59.9 ± 0.2 ^de^
21	0.23 ± 0.01 ^lmn^	2.64 ± 0.05 ^r^	2.88 ± 0.05 ^p^	14.1 ± 0.6 ^hijk^	15.10 ± 0.06 ^fgh^	45.3 ± 0.1 ^bc^	14.30 ± 0.02 ^hijk^	23.1 ± 0.1 ^nop^	17.3 ± 0.1 ^jkl^	59.6 ± 0.1 ^fg^
22	0.23 ± 0.01 ^lmn^	2.83 ± 0.03 ^q^	3.06 ± 0.04 ^o^	15.40 ± 0.06 ^bcd^	15.16 ± 0.08 ^fgh^	45.15 ± 0.05 ^bc^	15.0 ± 0.1 ^cd^	22.6 ± 0.1 ^rs^	17.2 ± 0.1 ^klm^	60.2 ± 0.1 ^c^
23	0.24 ± 0.01 ^jklmn^	2.85 ± 0.02 ^pq^	3.09 ± 0.01 ^no^	13.37 ± 0.05 ^lm^	15.6 ± 0.1 ^cde^	45.7 ± 0.1 ^a^	13.68 ± 0.05 ^mn^	23.2 ± 0.2 ^no^	17.5 ± 0.1 ^hij^	59.3 ± 0.2 ^h^
24	0.24 ± 0.01 ^jklmn^	2.92 ± 0.09 ^p^	3.2 ± 0.1 ^n^	14.79 ± 0.06 ^efg^	15.34 ± 0.04 ^efg^	45.13 ± 0.02 ^bc^	13.3 ± 0.1 ^opqr^	24.6 ± 0.1 ^hi^	17.0 ± 0.1 ^mno^	58.4 ± 0.1 ^kl^
25	0.22 ± 0.01 ^mn^	3.22 ± 0.04 ^o^	3.44 ± 0.03 ^m^	15.3 ± 0.1 ^cde^	15.82 ± 0.04 ^cd^	44.2 ± 0.2 ^fgh^	12.79 ± 0.03 ^t^	25.7 ± 0.1 ^d^	17.3 ± 0.1 ^jk^	57.0 ± 0.2 ^r^
26	0.26 ± 0.02 ^hijkl^	2.82 ± 0.08 ^q^	3.07 ± 0.06 ^o^	14.0 ± 0.1 ^hijk^	14.3 ± 0.5 ^kl^	43.6 ± 0.1 ^jkl^	13.02 ± 0.01 ^rst^	27.2 ± 0.3 ^a^	16.3 ± 0.5 ^t^	56.6 ± 0.1 ^s^
27	0.32 ± 0.01 ^bcd^	3.21 ± 0.04 ^oq^	3.53 ± 0.04 ^l^	13.6 ± 0.5 ^jklm^	14.45 ± 0.06 ^jkl^	45.1 ± 0.1 ^bc^	13.38 ± 0.07 ^nop^	25.0 ± 0.2 ^efg^	16.6 ± 0.1 ^rs^	58.5 ± 0.2 ^kl^
28	0.21 ± 0.01 ^mn^	2.85 ± 0.09 ^pq^	3.06 ± 0.08 ^o^	13.1 ± 0.4 ^mn^	14.7 ± 0.1 ^ijk^	44.33 ± 0.01 ^efg^	13.4 ± 0.4 ^opq^	24.7 ± 0.3 ^ghi^	17.6 ± 0.1 ^gh^	57.7 ± 0.4 ^no^
29	0.27 ± 0.01 ^ghij^	2.79 ± 0.04 ^q^	2.79 ± 0.04 ^o^	12.63 ± 0.09 ^n^	15.3 ± 0.1 ^efg^	45.34 ± 0.04 ^abc^	14.6 ± 0.1 ^efgh^	20.5 ± 0.2 ^v^	19.5 ± 0.1 ^c^	59.9 ± 0.1 ^de^
30	0.23 ± 0.06 ^mn^	3.33 ± 0.06 ^n^	3.6 ± 0.1 ^kl^	14.4 ± 0.5 ^ghi^	15.3 ± 0.4 ^efg^	43.88 ± 0.09 ^hij^	13.54 ± 0.09 ^mno^	23.4 ± 0.5 ^n^	19.2 ± 0.5 ^d^	57.4 ± 0.1 ^pq^
31	0.28 ± 0.01 ^fgh^	3.34 ± 0.05 ^n^	3.62 ± 0.06 ^k^	13.07 ± 0.01 ^mn^	17.4 ± 0.2 ^b^	44.3 ± 0.3 ^fg^	12.9 ± 0.2 ^st^	22.8 ± 0.1 ^qrs^	20.0 ± 0.3 ^b^	57.2 ± 0.1 ^q^
32	0.28 ± 0.04 ^fgh^	3.60 ± 0.03 ^l^	3.60 ± 0.01 ^i^	14.2 ± 0.2 ^hi^	15.50 ± 0.02 ^def^	44.5 ± 0.26 ^ef^	13.3 ± 0.5 ^opqr^	22.6 ± 0.2 ^st^	19.6 ± 0.1 ^c^	57.8 ± 0.2 ^n^
33	0.23 ± 0.01 ^klmn^	3.56 ± 0.05 ^lm^	3.80 ± 0.04 ^j^	15.7 ± 0.5 ^abc^	19.6 ± 0.2 ^a^	40.0 ± 0.3 ^o^	13.1 ± 0.1 ^qrst^	25.9 ± 0.6 ^cd^	21.0 ± 0.2 ^a^	53.1 ± 0.4 ^t^

^1^ C16:0—palmitic acid; C18:1n9—oleic acid; C18:2n6—linoleic acid; C18:3n3—α-linolenic acid; SFA—saturated fatty acids; MUFA—monounsaturated fatty acids; PUFA—polyunsaturated fatty acids. Different letters in the same column show significant difference between means according to Tukey’s HSD test (*p* < 0.05). * *Lens culinaris*, sub-species microsperma, Figure [43] and the levels of low-density lipoprotein (LDL) in blood [44], respectively. In addition, PUFA have shown accountable cellular purposes such as signaling, cell membrane fluidity, and structural conservation. They also control the nervous system, blood pressure, hematic clotting, glucose tolerance, and inflammatory processes, which may be beneficial in all inflammatory situations [45].

**Table 5:** Retention time (Rt), wavelengths of maximum absorption in the visible region (λ_max_), mass spectral data and identification of the phenolic compounds present in the Pardina lentil “Lenteja de Tierra de Campos”.

Peak	Rt (min)	λ_max_ (nm)	[M-H]^−^ (*m*/*z*)	MS^2^ (*m*/*z*)	Tentative identification
1	11.26	347	901	593(35), 285(75)	Kaempferol-deoxyhexoside-hexoside isomer I
2	11.97	347	901	593(35), 285(75)	Kaempferol-deoxyhexoside-hexoside isomer II
3	14.53	345	755	593(29), 285(100)	Kaempferol-*O*-hexoside-*O*-deoxyhexoside-hexoside

**Table 6:** Phenolic compounds (μg/100 g fw) of the studied Pardina lentil “Lenteja de Tierra de Campos” (mean ± SD, *n* = 3).

Sample *^1^	Kaempferol- Deoxyhexoside- Hexoside	Kaempferol-Deoxyhexoside-Hexoside	Kaempferol-*O*-Hexoside-*O*- Deoxyhexoside-Hexoside	Total Phenolic Compounds
1	29.6 ± 0.1 ^u^	25.6 ± 0.2 ^lm^	23.2 ± 0.1 ^o^	80 ± 0.01 ^v^
2	29.0 ± 0.1 ^v^	26.1 ± 0.4 ^k^	24.9 ± 0.1 ^d^	80 ± 0.01 ^u^
3	38.8 ± 0.5 ^s^	28.7 ± 0.1 ^e^	23.5 ± 0.1 ^n^	90 ± 0.01 ^p^
4	40.7 ± 0.9 ^o^	30.1 ± 0.9 ^c^	23.7 ± 0.1 ^jklmn^	90 ± 0.01 ^l^
5	25.6 ± 0.1 ^y^	nd	nd	30 ± 0.01 ^z^
6	39.0 ± 0.1 ^rs^	28.5 ± 0.1 ^e^	23.6 ± 0.1 ^mn^	90 ± 0.01 ^p^
7	40.8 ± 0.1 ^o^	29.1 ± 0.5 ^d^	23.6 ± 0.1 ^mn^	90 ± 0.01 ^m^
8	39.5 ± 0.1 5 ^pq^	28.7 ± 0.1 ^e^	23.5 ± 0.1 ^n^	90 ± 0.01 ^o^
9	23.0 ± 0. 1^z^	24.2 ± 0.1 ^q^	23.0 ± 0.1 ^o^	70 ± 0.01 ^x^
10	58.1 ± 0.1 ^c^	34.6 ± 0.1 ^a^	24.0 ± 0.1 ^fg^	120 ± 0.01 ^c^
11	35.0 ± 0.1 ^t^	35.0 ± 0.1 ^j^	35.0 ± 0.1 ^d^	90 ± 0.01 ^s^
12	23.0 ± 0.1 ^z^	23.0 ± 0.1 ^q^	23.0 ± 0.1 ^o^	70 ± 0.01 ^x^
13	41.3 ± 0.1 ^n^	41.3 ± 0.3 ^f^	41.3 ± 0.1 ^e^	90 ± 0.01 ^m^
14	28.2 ± 0.2 ^x^	28.2 ± 0.1 ^r^	28.2 ± 0.2 ^b^	80 ± 0.01 ^t^
15	39.3 ± 0.5 ^qr^	39.3 ± 0.4 ^j^	39.3 ± 0.1 ^ghij^	90 ± 0.01 ^q^
16	43.1 ± 0.1 ^l^	43.1 ± 0.2 ^fg^	43.1 ± 0.1 ^fgh^	100 ± 0.01 ^jk^
17	52.3 ± 0.1 ^e^	52.3 ± 0.2 ^b^	52.3 ± 0.1 ^ghijk^	110 ± 0.01 ^f^
18	50.8 ± 0.1 ^f^	50.8 ± 0.1 ^gh^	50.8 ± 0.1 ^ghi^	100 ± 0.01 ^g^
19	48.1 ± 0.2 ^h^	48.1 ± 0.6 ^e^	48.1 ± 0.1 ^ghi^	100 ± 0.01 ^g^
20	56.3 ± 0.1 ^d^	56.3 ± 0.1 ^c^	56.3 ± 0.1 ^ghi^	110 ± 0.01 ^d^
21	71.6 ± 0.7 ^b^	71.6 ± 0.1 ^s^	71.6 ± 0.7 ^c^	120 ± 0.01 ^b^
22	57.9 ± 0.1 ^c^	57.9 ± 0.4 ^i^	57.9 ± 0.1 ^d^	110 ± 0.01 ^e^
23	43.9 ± 0.2 ^k^	43.9 ± 0.2 ^h^	43.9 ± 0.1 ^ghij^	100 ± 0.01 ^jk^
24	44.9 ± 0.2 ^j^	44.9 ± 0.2 ^j^	44.9 ± 0.1 ^ef^	100 ± 0.01 ^jk^
25	42.6 ± 0.3 ^m^	42.6 ± 0.1 ^kl^	42.6 ± 0.1 ^fgh^	90 ± 0.01 ^n^
26	48.1 ± 0.2 ^h^	28.7 ± 0.2 ^e^	76.8 ± 0.8 ^a^	150 ± 0.01 ^a^
27	46.6 ± 0.1 ^i^	46.6 ± 0.1 ^no^	46.6 ± 0.1 ^hijkl^	100 ± 0.01 ^j^
28	157.8 ± 0.2 ^a^	157.8 ± 0.2 ^op^	157.8 ± 0.1 ^ijklm^	50 ± 0.01 ^y^
29	45.0 ± 0.1 ^j^	45.0 ± 0.1 ^p^	45.0 ± 0.1 ^ghijk^	90 ± 0.01 ^m^
30	43.9 ± 0.1 ^k^	43.9 ± 0.2 ^op^	43.9 ± 0.1 ^ghijk^	90 ± 0.01 ^n^
31	46.4 ± 0.1 ^i^	46.4 ± 0.1 ^nop^	46.4 ± 0.1 ^lmn^	100 ± 0.01 ^jk^
32	48.4 ± 0.3 ^h^	48.4 ± 0.3 ^no^	48.4 ± 0.1 ^klmn^	100 ± 0.01 ^i^
33	49.5 ± 0.1 ^g^	49.5 ± 0.1 ^mn^	49.5 ± 0.1 ^ghijk^	100 ± 0.01 ^h^
34	39.7 ± 0.1 ^p^	39.7 ± 0.1 ^q^	39.7 ± 0.1 ^mn^	90 ± 0.01 ^r^

^1^ Different letters in the same column show significant differences between means according to Tukey’s HSD test (*p* < 0.05). * *Lens culinaris*, sub-species microsperma, family Europeae. nd- not detected.

**Table 7:** Antioxidant activity of the studied Pardina lentil “Lenteja de Tierra de Campos” (mean ± SD; *n* = 3).

Sample *^1^	OxHLIA (IC_50_ Values, µg/mL)		TBARS
	Δ*t* = 60 min	Δ*t* = 120 min	(EC_50_ Values, μg/mL)
1	249 ± 7 ^e^	629 ± 7 ^c^	219 ± 9 ^fghijk^
2	132 ± 6 ^mn^	260 ± 12^lmno^	211 ± 3 ^nopq^
3	211 ± 12 ^fgh^	904 ± 23 ^b^	217 ± 4 ^ghijklmn^
4	147 ± 6 ^klm^	243 ± 11 ^mnop^	215 ± 9 ^ghijklm^
5	196 ± 4 ^ghi^	329 ± 6 ^hij^	215 ± 9 ^hijklmn^
6	809 ± 22 ^a^	1312 ± 27 ^a^	219 ± 1 ^efghij^
7	303 ± 12 ^d^	522 ± 20 ^d^	213 ± 5 ^lmno^
8	298 ± 5 ^d^	453 ± 6 ^f^	219 ± 7 ^hijklmn^
9	222 ± 6 ^efg^	358 ± 10 ^gh^	214 ± 3 ^ijklmn^
10	236 ± 8 ^ef^	384 ± 14 ^g^	219 ± 8 ^efghij^
11	346 ± 10 ^bc^	582 ± 18 ^c^	214 ± 6 ^jklmn^
12	181 ± 11 ^hij^	345 ± 20 ^ghi^	216 ± 7 ^hijklmn^
13	95 ± 5	184 ± 10 ^q^	216 ± 4 ^hijklmn^
14	102 ± 5 ^n^	178 ± 9 ^q^	218 ± 3 ^ghijkl^
15	146 ± 6 ^klm^	253 ± 8 ^lmnop^	212 ± 8 ^mnop^
16	146 ± 3 ^klm^	269 ± 13 ^klmn^	211 ± 6 ^nopq^
17	176 ± 5 ^ijk^	301 ± 6 ^ijkl^	224 ± 5 ^abcdef^
18	244 ± 9 ^e^	392 ± 14 ^g^	220 ± 9 ^efghi^
19	291 ± 9 ^d^	467 ± 12 ^ef^	228 ± 4 ^a^
20	132 ± 7 ^mn^	316 ± 10 ^hijk^	228 ± 8 ^a^
21	143 ± 4 ^lm^	237 ± 7 ^nop^	220 ± 5 ^efghi^
22	187 ± 8 ^hi^	329 ± 11 ^hij^	225 ± 7 ^abcde^
23	193 ± 8 ^ghi^	317 ± 13 ^hijk^	220 ± 3 ^defgh^
24	131 ± 4 ^mn^	288 ± 3 ^jklm^	226 ± 2 ^abcd^
25	85 ± 4	204 ± 8 ^pq^	222 ± 3 ^bcdefg^
26	63 ± 4	214 ± 11 ^opq^	222 ± 5 ^bcdefg^
27	169 ± 6 ^ijkl^	290 ± 9 ^jklm^	226 ± 4 ^abc^
28	244 ± 7 ^e^	428 ± 15 ^f^	227 ± 4 ^ab^
29	321 ± 12 ^cd^	519 ± 21 ^de^	213 ± 4 ^klmno^
30	362 ± 12 ^b^	587 ± 21 ^c^	221 ± 8 ^cdefgh^
31	142 ± 8 ^b^	253 ± 16 ^c^	206 ± 6 ^q^
32	152 ± 4 ^lm^	300 ± 6 ^lmnop^	212 ± 3 ^mnop^
33	140 ± 5 ^jklm^	260 ± 7 ^ijkl^	206 ± 1 ^pq^
34	145 ± 6 ^klm^	288 ± 9 ^jklm^	208 ± 1 ^opq^

^1^ Different letters in the same column show significant difference between means according to Tukey’s HSD test (*p* < 0.05). * *Lens culinaris*, sub-species microsperma, family Europeae.

## Appendix A

**Table A1:** Detailed fatty acid composition (%) of the studied Pardina lentil “Lenteja de Tierra de Campos” (mean ± SD).

	**1**	**2**	**3**	**4**	**5**	**6**	**7**	**8**	**9**	**10**
C6:0	0.68 ± 0.06	0.15 ± 0.015	0.32 ± 0.01	0.07 ± 0.004	0.759 ± 0.001	0.79 ± 0.04	0.93 ± 0.04	0.20 ± 0.01	0.12 ± 0.01	0.21 ± 0.01
C8:0	0.13 ± 0.01	0.03 ± 0.01	0.08 ± 0.01	0.03 ± 0.01	0.09 ± 0.01	0.07 ± 0.01	0.05 ± 0.01	0.02 ± 0.01	0.03 ± 0.01	0.10 ± 0.01
C11:0	0.09 ± 0.008	0.091 ± 0.008	0.11 ± 0.01	0.028 ± 0.001	0.14 ± 0.01	0.043 ± 0.001	0.025 ± 0.001	0.037 ± 0.003	0.07 ± 0.01	0.054 ± 0.004
C12:0	0.10 ± 0.004	0.076 ± 0.002	0.09 ± 0.01	0.039 ± 0.003	0.09 ± 0.01	0.09 ± 0.01	0.09 ± 0.01	0.040 ± 0.002	0.04 ± 0.01	0.08 ± 0.01
C13:0	0.09 ± 0.01	0.07 ± 0.01	0.06 ± 0.01	0.15 ± 0.01	0.09 ± 0.01	0.08 ± 0.01	0.08 ± 0.01	0.08 ± 0.01	0.12 ± 0.01	0.27 ± 0.01
C14:0	0.75 ± 0.03	0.76 ± 0.01	0.531 ± 0.003	0.61 ± 0.01	0.38 ± 0.03	0.57 ± 0.06	0.50 ± 0.03	0.45 ± 0.01	0.43 ± 0.01	0.352 ± 0.004
C15:0	0.77 ± 0.02	0.37 ± 0.01	0.55 ± 0.01	0.36 ± 0.03	0.63 ± 0.01	0.30 ± 0.01	0.44 ± 0.03	0.44 ± 0.01	0.39 ± 0.01	0.80 ± 0.07
C16:0	14.14 ± 0.01	15.93 ± 0.04	14.31 ± 0.02	15.3 ± 0.3	14.2 ± 0.2	15.24 ± 0.07	14.0 ± 0.1	15.5 ± 0.3	16.1 ± 0.1	15.37 ± 0.09
C16:1	0.13 ± 0.01	0.140 ± 0.004	0.18 ± 0.01	0.12 ± 0.01	0.29 ± 0.01	0.38 ± 0.01	0.47 ± 0.01	0.41 ± 0.01	0.88 ± 0.01	0.90 ± 0.01
C17:0	0.40 ± 0.01	0.289 ± 0.004	0.34 ± 0.01	0.32 ± 0.01	0.63 ± 0.01	0.82 ± 0.05	0.9 ± 0.1	0.14 ± 0.01	0.04 ± 0.01	0.04 ± 0.01
C18:0	2.59 ± 0.17	1.95 ± 0.01	2.4 ± 0.2	2.4 ± 0.2	1.41 ± 0.01	2.4 ± 0.2	2.1 ± 0.1	2.27 ± 0.01	2.18 ± 0.01	2.11 ± 0.06
C18:1n9c	15.12 ± 0.04	14.08 ± 0.02	14.2 ± 0.2	15.7 ± 0.2	16.0 ± 0.1	14.3 ± 0.1	14.15 ± 0.05	13.65 ± 0.07	13.08 ± 0.08	14.7 ± 0.3
C18:2n6c	44.08 ± 0.06	45.13 ± 0.01	44.52 ± 0.01	44.2 ± 0.2	43.8 ± 0.2	43.30 ± 0.04	42.76 ± 0.09	43.99 ± 0.02	45.27 ± 0.06	43.4 ± 0.2
C18:3n3	14.11 ± 0.07	14.84 ± 0.07	15.41 ± 0.04	14.24 ± 0.06	13.19 ± 0.06	14.16 ± 0.04	15.42 ± 0.03	15.37 ± 0.01	14.43 ± 0.01	14.8 ± 0.4
C20:0	1.5 ± 0.2	1.35 ± 0.03	1.06 ± 0.07	1.47 ± 0.08	2.20 ± 0.06	1.69 ± 0.02	1.69 ± 0.09	1.85 ± 0.03	1.49 ± 0.07	1.61 ± 0.03
C20:1	1.05 ± 0.07	0.99 ± 0.02	0.92 ± 0.01	1.00 ± 0.04	1.2 ± 0.1	1.38 ± 0.07	1.6 ± 0.1	1.7 ± 0.1	1.54 ± 0.02	1.27 ± 0.03
C21:0	0.70 ± 0.07	0.84 ± 0.08	0.95 ± 0.04	0.60 ± 0.02	0.77 ± 0.02	0.91 ± 0.06	0.42 ± 0.04	0.18 ± 0.01	0.15 ± 0.01	0.22 ± 0.01
C22:0	1.84 ± 0.08	1.26 ± 0.01	1.51 ± 0.01	1.40 ± 0.08	1.4 ± 0.1	1.29 ± 0.09	1.49 ± 0.03	1.07 ± 0.01	1.01 ± 0.01	1.21 ± 0.03
C22:1	0.29 ± 0.02	0.25 ± 0.01	0.34 ± 0.02	0.22 ± 0.01	0.39 ± 0.01	0.28 ± 0.01	0.52 ± 0.03	0.83 ± 0.01	0.85 ± 0.01	0.84 ± 0.04
C23:0	0.21 ± 0.01	0.33 ± 0.02	0.53 ± 0.03	0.70 ± 0.03	0.39 ± 0.01	0.56 ± 0.01	0.75 ± 0.03	1.01 ± 0.07	0.94 ± 0.05	0.95 ± 0.05
C24:0	1.19 ± 0.09	1.07 ± 0.07	1.60 ± 0.06	1.1 ± 0.1	1.96 ± 0.05	1.33 ± 0.05	1.6 ± 0.1	0.83 ± 0.01	0.88 ± 0.04	0.77 ± 0.06
SFA	25.2 ± 0.1	24.6 ± 0.1	24.5 ± 0.1	24.6 ± 0.4	25.2 ± 0.3	26.2 ± 0.1	25.1 ± 0.1	24.1 ± 0.1	23.9 ± 0.1	24.1 ± 0.3
MUFA	16.6 ± 0.1	24.6 ± 0.1	15.6 ± 0.1	17.0 ± 0.2	17.9 ± 0.1	16.4 ± 0.2	16.8 ± 0.1	16.6 ± 0.2	16.4 ± 0.1	17.7 ± 0.3
PUFA	58.2 ± 0.1	60.0 ± 0.1	59.9 ± 0.1	58.5 ± 0.2	57.0 ± 0.3	57.5 ± 0.1	58.2 ± 0.1	59.4 ± 0.1	59.7 ± 0.1	58.2 ± 0.6
	**11**	**12**	**13**	**14**	**15**	**16**	**17**	**18**	**19**	**20**
C6:0	0.057 ± 0.001	0.094 ± 0.004	0.087 ± 0.005	0.091 ± 0.002	0.068 ± 0.004	0.082 ± 0.008	0.075 ± 0.006	0.073 ± 0.001	0.044 ± 0.002	0.055 ± 0.004
C8:0	0.054 ± 0.001	0.068 ± 0.004	0.08 ± 0.01	0.09 ± 0.01	0.08 ± 0.01	0.04 ± 0.01	0.15 ± 0.01	0.26 ± 0.01	0.03 ± 0.01	0.04 ± 0.01
C11:0	0.034 ± 0.002	0.071 ± 0.004	0.032 ± 0.001	0.062 ± 0.004	0.033 ± 0.002	0.065 ± 0.001	0.092 ± 0.004	0.061 ± 0.003	0.07 ± 0.01	0.08 ± 0.01
C12:0	0.037 ± 0.004	0.042 ± 0.002	0.042 ± 0.002	0.07 ± 0.01	0.063 ± 0.006	0.066 ± 0.003	0.08 ± 0.01	0.097 ± 0.006	0.07 ± 0.01	0.064 ± 0.002
C13:0	0.13 ± 0.01	0.13 ± 0.01	0.174 ± 0.002	0.09 ± 0.01	0.21 ± 0.01	0.27 ± 0.01	0.39 ± 0.02	0.37 ± 0.02	0.21 ± 0.02	0.19 ± 0.01
C14:0	0.39 ± 0.04	0.40 ± 0.02	0.59 ± 0.02	0.35 ± 0.03	0.72 ± 0.02	0.09 ± 0.01	0.08 ± 0.01	0.092 ± 0.001	0.62 ± 0.03	0.35 ± 0.01
C15:0	0.35 ± 0.02	0.36 ± 0.01	0.42 ± 0.01	0.37 ± 0.03	0.55 ± 0.04	0.68 ± 0.03	0.17 ± 0.01	0.111 ± 0.002	0.32 ± 0.01	0.33 ± 0.02
C16:0	15.17 ± 0.07	14.96 ± 0.04	13.42 ± 0.09	14.2 ± 0.3	15.4 ± 0.2	15.9 ± 0.1	14.5 ± 0.5	15.0 ± 0.2	13.9 ± 0.2	13.99 ± 0.01
C16:1	0.69 ± 0.06	0.65 ± 0.01	0.66 ± 0.01	0.48 ± 0.01	0.54 ± 0.01	0.33 ± 0.03	0.44 ± 0.02	0.61 ± 0.01	0.45 ± 0.01	0.23 ± 0.02
C17:0	0.50 ± 0.01	0.48 ± 0.01	0.55 ± 0.01	0.50 ± 0.01	0.09 ± 0.01	0.34 ± 0.01	0.14 ± 0.01	0.79 ± 0.02	0.67 ± 0.01	0.17 ± 0.01
C18:0	2.10 ± 0.06	2.03 ± 0.01	2.53 ± 0.03	2.5 ± 0.2	2.1 ± 0.1	2.05 ± 0.02	2.41 ± 0.11	2.21 ± 0.01	2.05 ± 0.02	2.28 ± 0.09
C18:1n9c	14.49 ± 0.07	14.2 ± 0.1	15.29 ± 0.05	15.44 ± 0.06	14.97 ± 0.02	14.95 ± 0.08	14.8 ± 0.2	13.3 ± 0.2	14.4 ± 0.1	15.4 ± 0.6
C18:2n6c	44.7 ± 0.3	45.1 ± 0.2	44.17 ± 0.02	43.45 ± 0.03	44.2 ± 0.3	43.8 ± 0.3	44.11 ± 0.01	44.5 ± 0.3	44.99 ± 0.08	45.4 ± 0.1
C18:3n3	15.74 ± 0.05	15.85 ± 0.03	14.60 ± 0.03	15.25 ± 0.05	13.8 ± 0.1	14.76 ± 0.05	14.16 ± 0.07	14.4 ± 0.1	14.63 ± 0.03	14.48 ± 0.04
C20:0	0.88 ± 0.08	0.89 ± 0.04	1.22 ± 0.08	1.07 ± 0.02	1.04 ± 0.03	1.02 ± 0.01	1.4 ± 0.1	1.05 ± 0.09	0.43 ± 0.02	0.88 ± 0.01
C20:1	1.3 ± 0.1	1.41 ± 0.02	1.76 ± 0.04	1.66 ± 0.12	1.27 ± 0.05	1.6 ± 0.1	1.40 ± 0.03	1.75 ± 0.06	1.7 ± 0.2	1.11 ± 0.01
C21:0	0.60 ± 0.01	0.72 ± 0.01	0.69 ± 0.04	0.42 ± 0.02	0.93 ± 0.06	0.2 ± 0.1	0.66 ± 0.02	0.70 ± 0.03	1.25 ± 0.03	1.39 ± 0.07
C22:0	0.29 ± 0.01	0.335 ± 0.003	1.15 ± 0.07	1.11 ± 0.01	1.01 ± 0.01	1.25 ± 0.02	1.22 ± 0.01	1.015 ± 0.004	1.29 ± 0.08	1.12 ± 0.05
C22:1	0.72 ± 0.02	0.61 ± 0.01	0.74 ± 0.02	0.81 ± 0.03	0.80 ± 0.06	0.24 ± 0.02	0.35 ± 0.01	0.52 ± 0.01	0.8 ± 0.1	0.58 ± 0.03
C23:0	0.95 ± 0.03	0.87 ± 0.05	0.89 ± 0.01	0.98 ± 0.03	1.02 ± 0.02	1.14 ± 0.04	1.71 ± 0.03	1.6 ± 0.1	1.02 ± 0.07	0.95 ± 0.06
C24:0	0.82 ± 0.05	0.79 ± 0.08	0.91 ± 0.06	1.0 ± 0.1	1.08 ± 0.01	1.12 ± 0.01	1.70 ± 0.01	1.48 ± 0.04	1.05 ± 0.06	0.90 ± 0.04
SFA	22.4 ± 0.1	22.2 ± 0.3	22.8 ± 0.1	22.9 ± 0.1	24.4 ± 0.3	24.3 ± 0.1	24.8 ± 0.3	24.9 ± 0.2	23.0 ± 0.1	22.8 ± 0.3
MUFA	17.2 ± 0.1	16.8 ± 0.1	18.4 ± 0.1	18.4 ± 0.1	17.6 ± 0.1	17.1 ± 0.1	17.0 ± 0.2	16.2 ± 0.1	17.4 ± 0.1	17.3 ± 0.5
PUFA	60.4 ± 0.2	60.9 ± 0.2	58.8 ± 0.1	58.7 ± 0.1	58.1 ± 0.4	58.6 ± 0.2	58.3 ± 0.1	58.9 ± 0.1	59.6 ± 0.1	59.9 ± 0.2
	**21**	**22**	**23**	**24**	**25**	**26**	**27**	**28**	**29**	**30**
C6:0	0.035 ± 0.003	0.074 ± 0.004	0.106 ± 0.001	0.18 ± 0.01	0.062 ± 0.001	0.17 ± 0.01	0.17 ± 0.01	0.03 ± 0.01	0.06 ± 0.01	0.62 ± 0.02
C8:0	0.092 ± 0.003	0.03 ± 0.01	0.03 ± 0.01	0.04 ± 0.01	0.03 ± 0.01	0.38 ± 0.01	0.04 ± 0.01	0.04 ± 0.01	0.02 ± 0.01	0.40 ± 0.01
C11:0	0.037 ± 0.002	0.057 ± 0.003	0.082 ± 0.004	0.08 ± 0.01	0.066 ± 0.001	0.056 ± 0.003	0.16 ± 0.01	0.06 ± 0.01	0.03 ± 0.01	0.32 ± 0.01
C12:0	0.053 ± 0.004	0.06 ± 0.01	0.11 ± 0.01	0.043 ± 0.003	0.059 ± 0.003	0.082 ± 0.004	0.06 ± 0.01	0.09 ± 0.01	0.03 ± 0.01	0.14 ± 0.01
C13:0	0.079 ± 0.005	0.08 ± 0.01	0.16 ± 0.01	0.164 ± 0.004	0.22 ± 0.01	0.38 ± 0.03	0.21 ± 0.01	0.16 ± 0.01	0.11 ± 0.01	0.58 ± 0.03
C14:0	0.43 ± 0.01	0.47 ± 0.02	0.71 ± 0.01	0.45 ± 0.03	0.656 ± 0.004	1.03 ± 0.04	0.59 ± 0.01	0.60 ± 0.02	0.41 ± 0.03	0.48 ± 0.02
C15:0	0.339 ± 0.004	0.39 ± 0.01	0.45 ± 0.01	0.40 ± 0.02	0.572 ± 0.002	1.1 ± 0.1	0.49 ± 0.01	0.53 ± 0.02	0.33 ± 0.02	0.13 ± 0.01
C16:0	14.1 ± 0.6	15.40 ± 0.06	13.37 ± 0.05	14.79 ± 0.06	15.3 ± 0.1	14.0 ± 0.1	13.6 ± 0.5	13.1 ± 0.4	12.63 ± 0.09	14.4 ± 0.5
C16:1	0.57 ± 0.02	0.45 ± 0.04	0.77 ± 0.01	0.45 ± 0.04	0.29 ± 0.02	0.31 ± 0.01	0.26 ± 0.01	0.31 ± 0.03	0.36 ± 0.02	0.93 ± 0.02
C17:0	0.54 ± 0.02	0.37 ± 0.01	0.88 ± 0.04	0.87 ± 0.01	0.55 ± 0.03	0.84 ± 0.02	0.76 ± 0.06	0.50 ± 0.01	0.34 ± 0.02	0.89 ± 0.04
C18:0	2.69 ± 0.06	2.03 ± 0.03	2.82 ± 0.04	2.81 ± 0.03	2.02 ± 0.02	2.0 ± 0.1	2.2 ± 0.2	2.06 ± 0.04	1.94 ± 0.04	1.21 ± 0.06
C18:1n9c	15.10 ± 0.06	15.16 ± 0.08	15.6 ± 0.1	15.34 ± 0.04	15.82 ± 0.04	14.3 ± 0.5	14.45 ± 0.06	14.7 ± 0.1	15.3 ± 0.1	15.3 ± 0.4
C18:2n6c	45.3 ± 0.1	45.15 ± 0.05	45.7 ± 0.1	45.13 ± 0.02	44.2 ± 0.2	43.6 ± 0.1	45.1 ± 0.1	44.33 ± 0.01	45.34 ± 0.04	43.88 ± 0.09
C18:3n3	14.30 ± 0.02	15.0 ± 0.1	13.68 ± 0.05	13.3 ± 0.1	12.79 ± 0.03	13.02 ± 0.01	13.38 ± 0.07	13.4 ± 0.4	14.6 ± 0.1	13.54 ± 0.09
C20:0	0.63 ± 0.02	0.92 ± 0.01	1.14 ± 0.07	1.06 ± 0.01	1.38 ± 0.09	1.76 ± 0.16	1.49 ± 0.02	1.32 ± 0.02	1.04 ± 0.02	1.03 ± 0.02
C20:1	1.25 ± 0.03	0.40 ± 0.03	0.58 ± 0.05	0.58 ± 0.04	0.42 ± 0.03	0.65 ± 0.06	0.8 ± 0.1	1.73 ± 0.04	2.86 ± 0.02	2.56 ± 0.08
C21:0	1.8 ± 0.2	0.36 ± 0.03	1.02 ± 0.01	0.73 ± 0.02	0.58 ± 0.03	0.89 ± 0.08	1.09 ± 0.01	2.6 ± 0.1	1.02 ± 0.07	1.11 ± 0.01
C22:0	0.88 ± 0.01	0.88 ± 0.08	1.07 ± 0.03	1.08 ± 0.02	1.5 ± 0.1	1.62 ± 0.03	1.46 ± 0.02	1.3 ± 0.1	0.94 ± 0.02	0.88 ± 0.02
C22:1	0.40 ± 0.04	1.20 ± 0.07	0.49 ± 0.04	0.66 ± 0.05	0.80 ± 0.07	0.97 ± 0.05	1.08 ± 0.03	0.90 ± 0.04	0.97 ± 0.02	0.42 ± 0.01
C23:0	0.67 ± 0.01	0.78 ± 0.07	0.88 ± 0.02	1.01 ± 0.06	1.28 ± 0.07	1.52 ± 0.05	1.19 ± 0.08	1.11 ± 0.07	0.84 ± 0.03	0.52 ± 0.01
C24:0	0.80 ± 0.05	0.76 ± 0.06	0.37 ± 0.01	0.86 ± 0.08	1.46 ± 0.06	1.32 ± 0.01	1.48 ± 0.02	1.15 ± 0.05	0.79 ± 0.01	0.67 ± 0.02
SFA	23.1 ± 0.1	22.6 ± 0.1	23.2 ± 0.2	24.6 ± 0.1	25.7 ± 0.1	27.2 ± 0.3	25.0 ± 0.2	24.7 ± 0.3	20.5 ± 0.2	23.4 ± 0.5
MUFA	17.3 ± 0.1	17.2 ± 0.1	17.5 ± 0.1	17.0 ± 0.1	17.3 ± 0.1	16.3 ± 0.5	16.6 ± 0.1	17.6 ± 0.1	19.5 ± 0.1	19.2 ± 0.5
PUFA	59.6 ± 0.1	60.2 ± 0.1	59.3 ± 0.2	58.4 ± 0.1	57.0 ± 0.2	56.6 ± 0.1	58.5 ± 0.2	57.7 ± 0.4	59.9 ± 0.1	57.4 ± 0.1

C6:0—caproic acid; C8:0—caprylic acid; C11:0—undecaenoic acid; C12:0—lauric acid; C13:0—tridecanoic acid; C14:0—myristic acid; C15:0—pentadecanoic acid; C16:0—palmitic acid; C16:1—palmitoleic acid; C17:0—heptadecanoic acid; C18:0—stearic acid; C18:1n9c—oleic acid; C18:2n6c—linoleic acid; C18:3n3—linolenic acid; C20:0—arachidic acid; C20:1—eicosenoic acid; C21:0—heneicosanoic acid; C22:0—behenic acid; C22:1—erucic acid; C23:0—tricosanoic acid and C24:0—lignoceric acid; SFA—saturated fatty acids; MUFA—monounsaturated fatty acids; PUFA—polyunsaturated fatty acids.
